# Semi-Urgent Totally Extraperitoneal Repair for Inguinal Hernia Causing Insufficient Intraperitoneal Exchange in a Patient on Peritoneal Dialysis: A Case Report

**DOI:** 10.70352/scrj.cr.26-0057

**Published:** 2026-03-12

**Authors:** Yume Yabu, Yoshiro Imai, Yusuke Suzuki, Ryo Tanaka, Hiroki Hamamoto, Kosei Kimura, Mitsuhiro Asakuma, Hideki Tomiyama, Mitsuhiko Iwamoto, Sang-Woong Lee

**Affiliations:** Department of General and Gastroenterological Surgery, Osaka Medical and Pharmaceutical University, Takatsuki, Osaka, Japan

**Keywords:** inguinal hernia, intraperitoneal exchange, peritoneal dialysis, case report

## Abstract

**INTRODUCTION:**

Inguinal hernias are mechanical complications in patients undergoing peritoneal dialysis (PD) that may threaten the continuation of PD. Herein, we report a rare case in which PD could not be completed owing to outflow failure associated with a large inguinal hernia that was successfully managed with semi-urgent totally extraperitoneal (TEP) repair.

**CASE PRESENTATION:**

A 79-year-old man with a known inguinal hernia was diagnosed with chronic kidney disease. On day 8 after PD initiation, the inguinoscrotal swelling worsened, and the dialysate could not be adequately recovered, making continuation of PD challenging. CT revealed a small bowel within the hernia sac with subcutaneous edema and hydrocele, although the PD catheter tip remained in an appropriate intraperitoneal position without migration to the hernia sac. Because urgent restoration of PD was required, a semi-urgent TEP repair was performed, avoiding peritoneal incision to facilitate early PD resumption. PD was restarted on POD 1 with total dialysate volume of 2000 mL/day, which increased to 4000 mL/day on POD 2, and returned to the usual volume of 6000 mL/day by POD 6. No recurrence was observed at 6 months.

**CONCLUSIONS:**

Large inguinal hernia can cause severe PD outflow failure even without catheter migration. Semi-urgent TEP repair may allow early resumption of PD and help preserve this modality in selected patients.

## Abbreviations

HD
: hemodialysis
PD
: peritoneal dialysis
TAPP
: transabdominal preperitoneal
TEP
: totally extraperitoneal

## INTRODUCTION

Peritoneal dialysis (PD) is an established renal replacement therapy that offers greater flexibility than HD in terms of time and location. Unlike in-center HD, which necessitates visits to a medical facility thrice a week with substantial time commitment, PD can be performed at home with routine outpatient follow-up often limited to once or twice per month. This flexibility reduces disruptions in work, school, and daily life, thereby supporting the patient’s autonomy and QOL. However, because PD requires an intraperitoneal catheter and repeated intraperitoneal infusions, it carries risks such as peritonitis.^1,2)^ Additionally, sustained or recurrent increases in intra-abdominal pressure associated with dialysate dwell may predispose patients to abdominal wall complications, such as ventral and inguinal hernias.^3–7)^ These complications can impair PD efficiency and, in some cases, lead to discontinuation of PD.

In our patient, dialysate sequestration within the inguinal hernia sac resulted in insufficient intraperitoneal exchange and threatened the continuation of PD. Therefore, we performed semi-urgent TEP repair. To our knowledge, this is the first report of PD failure due to dialysate sequestration in an inguinal hernia sac that was successfully managed with semi-urgent TEP repair.

## CASE PRESENTATION

**Fig. 1:**
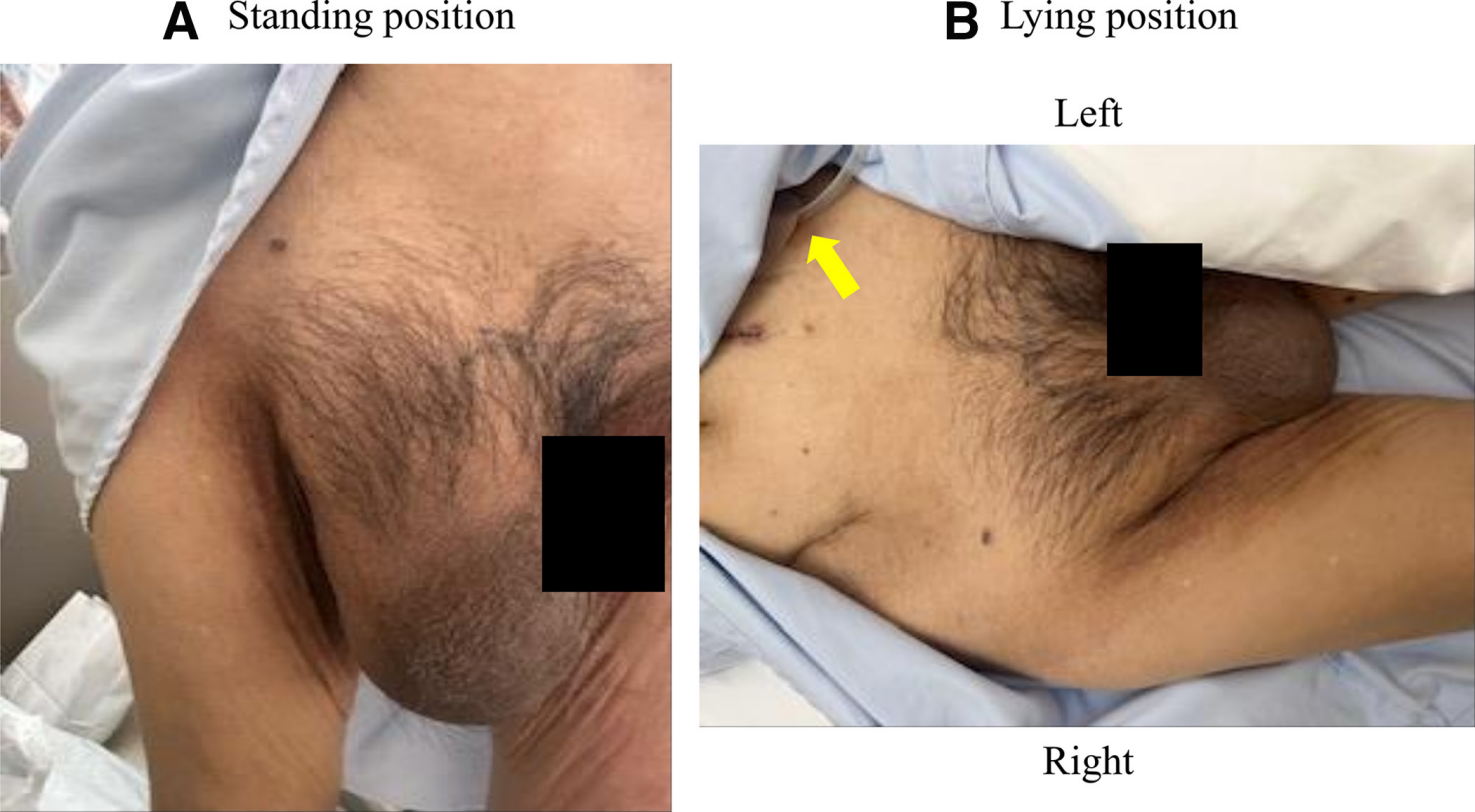
Physical examination findings. (**A**) Large inguinoscrotal swelling. (**B**) The PD catheter exit site on the left lateral abdominal wall (yellow arrow). PD, peritoneal dialysis

**Fig. 2:**
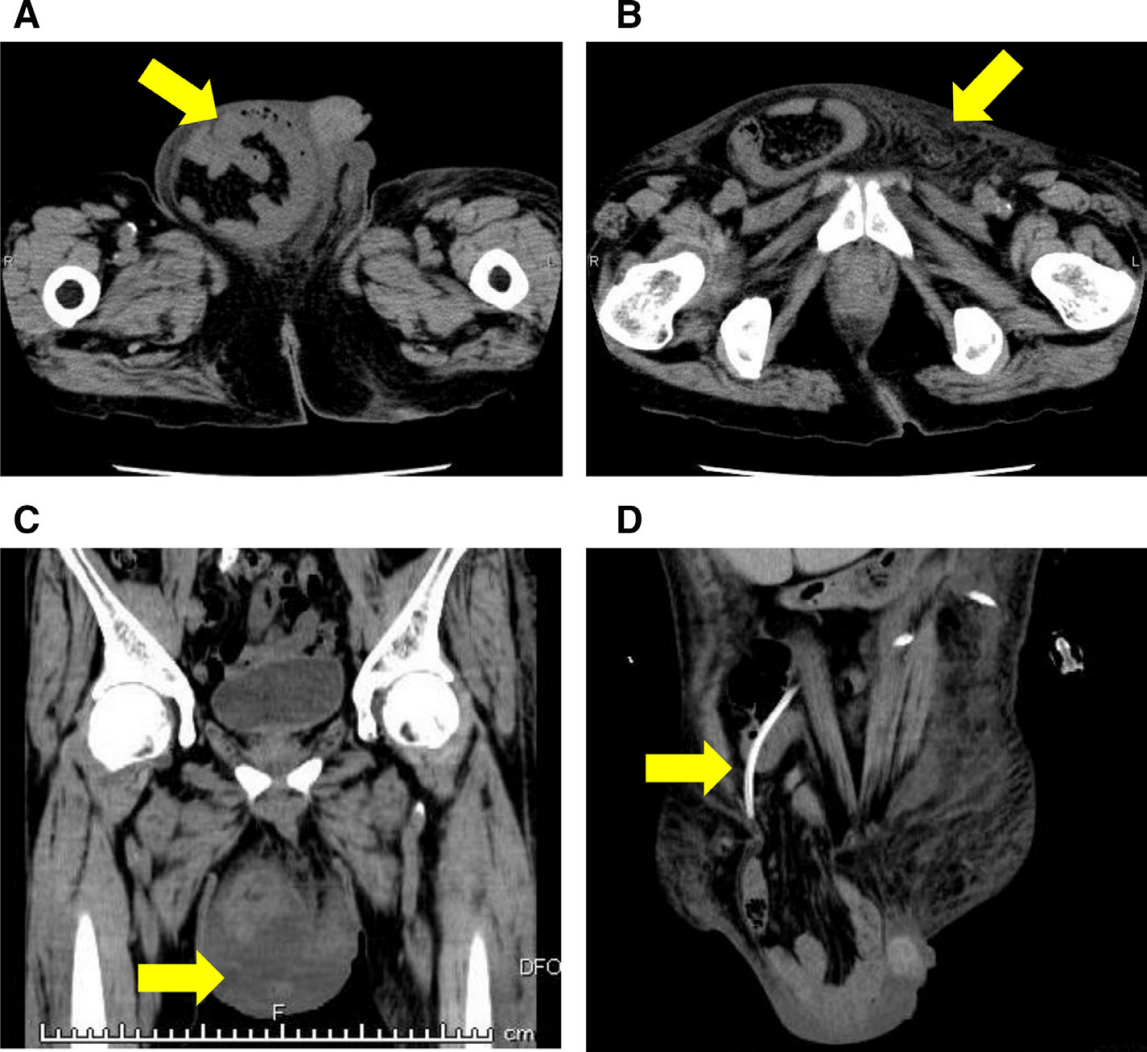
CT findings. (**A**) Small bowel within the inguinal hernia sac (yellow arrow). (**B**) Subcutaneous edema in the groin area (yellow arrow). (**C**) Hydrocele within the scrotum (yellow arrow). (**D**) The PD catheter tip remained in an appropriate intraperitoneal position without migration into the hernia sac (yellow arrow). PD, peritoneal dialysis

**Fig. 3:**
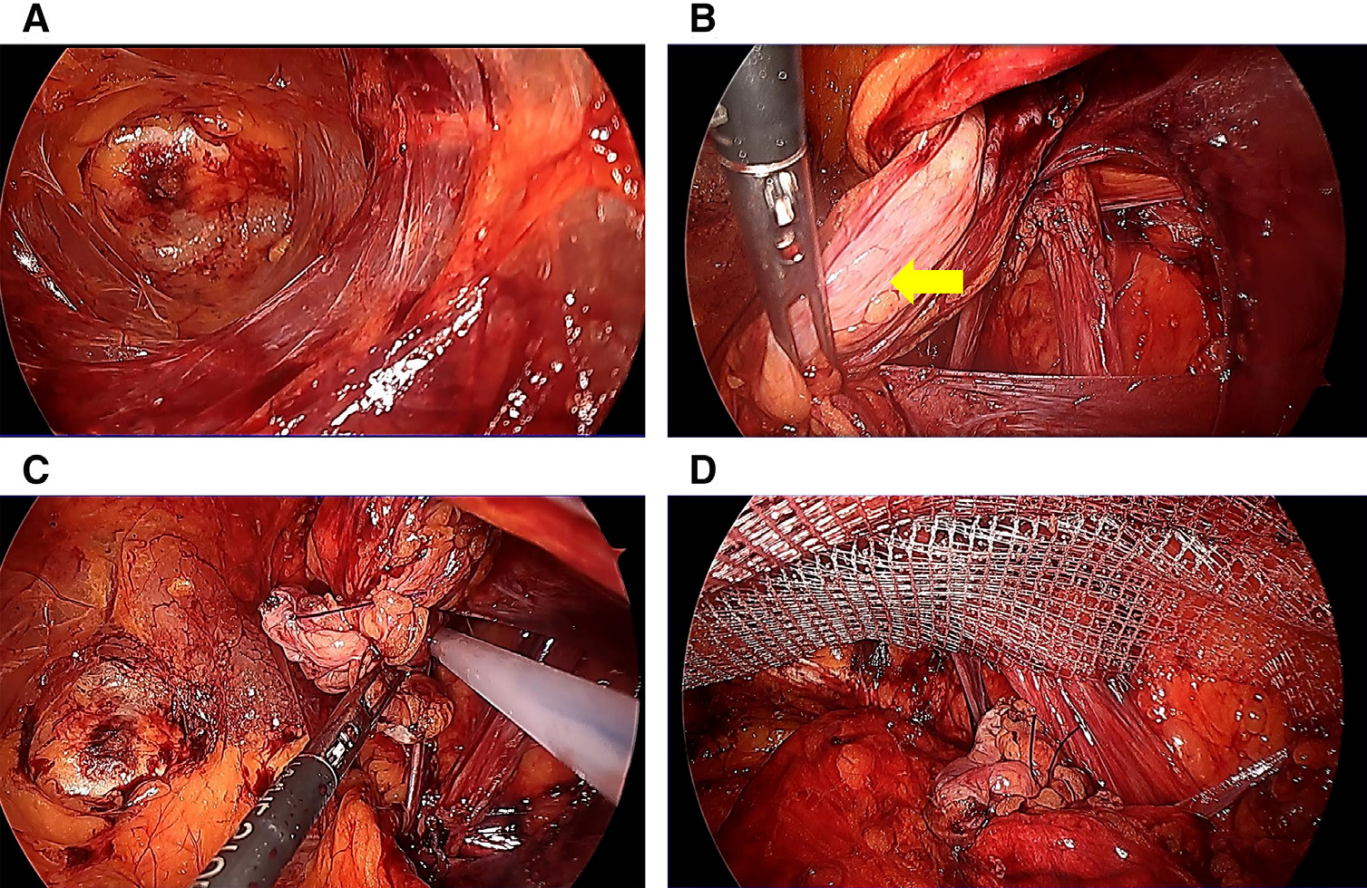
Intraoperative endoscopic view. (**A**) Marked edema in the preperitoneal space. (**B**) Hernia sac (yellow arrow). (**C**) Double ligation of the proximal stump using a pre-tied loop ligature (Endoloop PDS II; Ethicon). (**D**) Placement of a self-fixating mesh in the preperitoneal space (ProGrip; Medtronic).

**Fig. 4:**
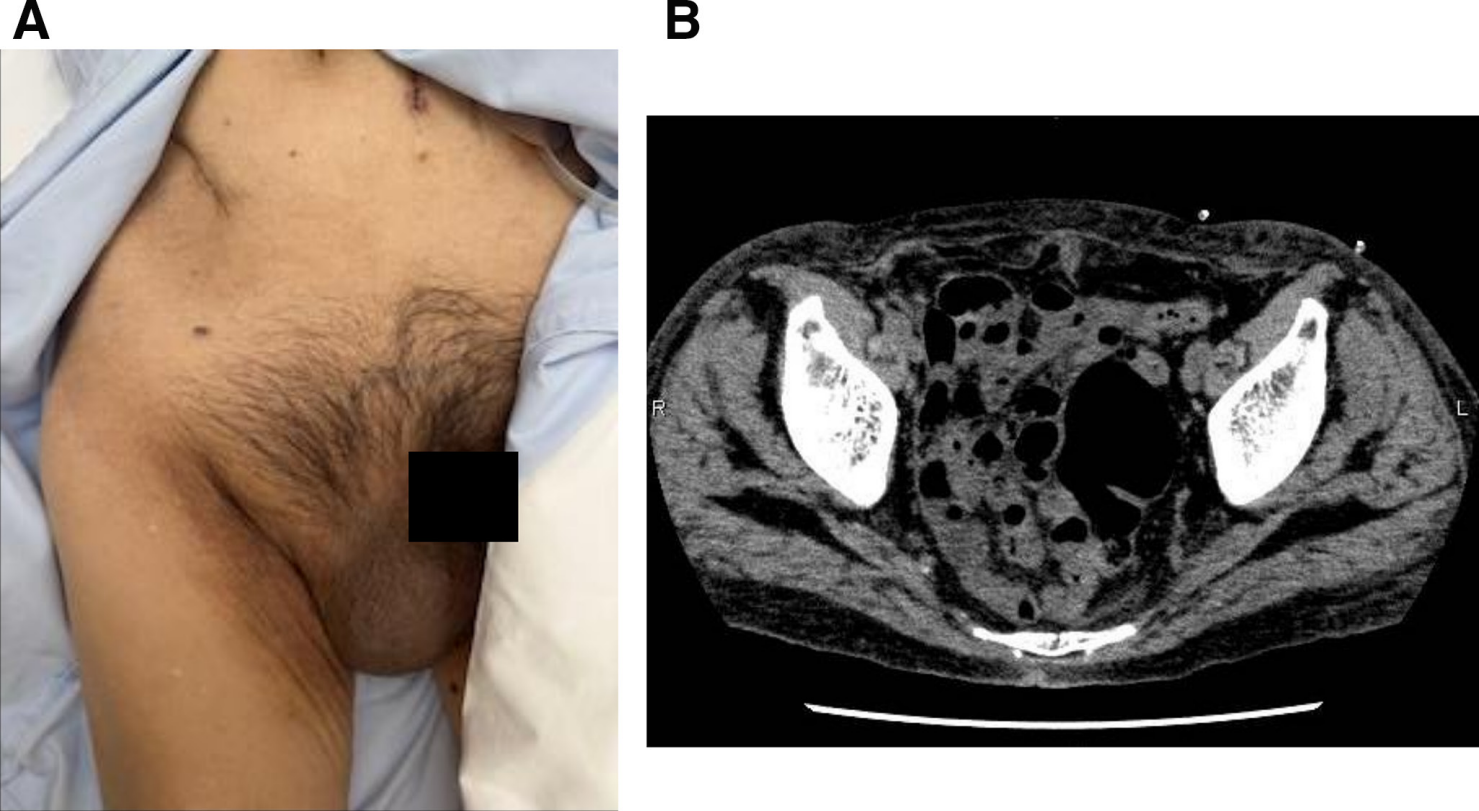
Postoperative findings at 6 months. (**A**) No inguinal swelling while standing. (**B**) No signs of recurrence on CT findings.

A 79-year-old man was previously diagnosed with an inguinal hernia and conservatively managed with observation because he was asymptomatic. He had not been referred to our surgical service before prior to PD initiation. Therefore, detailed clinical reasoning for observation was not available at that time. The patient was initiated on PD for chronic kidney disease. On day 8 after PD initiation, his inguinal bulge worsened, and the dialysate could no longer be adequately drained. Continuation of PD became difficult, and he was referred to our department for further evaluation and management the following day. Physical examination revealed that the inguinal swelling extended into the scrotum (**Fig. 1A**) and was reducible. The PD catheter exit site was located on the left lateral abdominal wall (**Fig. 1B**). CT revealed small bowel within the inguinal hernia sac (**Fig. 2A**), accompanied by subcutaneous edema (**Fig. 2B**) and a hydrocele (**Fig. 2C**). No migration of the PD catheter into the hernial sac was observed, and the catheter tip remained in the appropriate intraperitoneal position (**Fig. 2D**). Direct imaging confirmation was not available. Hernia-related impairment of dialysate exchange was considered an explanation owing to the worsening of the inguinal swelling after PD initiation and the absence of catheter migration on CT. To maintain PD, surgical repair of the inguinal hernia was considered as the only viable option. Considering early resumption of PD, we selected TEP repair, which does not require a peritoneal incision and allows for hernia correction without entering the peritoneal cavity. The operation was performed on the day following the preoperative evaluation. Intraoperatively, the preperitoneal space was markedly edematous (**Fig. 3A**), likely due to ongoing PD. The procedure was performed meticulously to avoid peritoneal injury. After identifying the hernial sac, we attempted dissection toward the distal end (**Fig. 3B**); however, because the sac was extremely large and had formed a scar tissue, we decided to transect it distally. The hernial sac was opened to confirm the absence of bowel contents, after which it was resected. The proximal stump was secured with double ligation using a pre-tied loop ligature (Endoloop PDS II; Ethicon, Cincinnati, OH, USA) (**Fig. 3C**). A self-fixating mesh (ProGrip; Medtronic, Dublin, Ireland) was placed in the preperitoneal space, and the procedure was concluded (**Fig. 3D**). PD was resumed on POD 1, with a total dialysate volume of 2000 mL. The volume increased to 4000 mL on POD 2, and by POD 5 it returned to the usual volume of 6000 mL. Six months postoperatively, there were no clinical (**Fig. 4A**) or radiological signs of recurrence (**Fig. 4B**).

## DISCUSSION

This case report highlights a rare but clinically important mechanism of PD dysfunction in a patient with a pre-existing large inguinal hernia. In our patient, dialysate preferentially accumulated within the markedly enlarged inguinal hernia sac resulting in insufficient intraperitoneal exchange and failure to recover the dialysate, despite the PD catheter remaining in an appropriate position on CT. Consequently, the continuation of PD was not feasible without addressing the hernia. Although hernias during PD are well-recognized and may lead to dialysate leakage, genital swelling, or technique compromise, reports describing severe PD failure attributable to dialysate sequestration within a giant inguinal hernia sac without catheter migration are scarce.^8)^ From a practical standpoint, this case underscores that patients with known inguinal hernias, particularly large or progressive hernias, require careful evaluation when PD is planned or initiated. Abdominal wall integrity is a prerequisite for successful PD, and routine screening and appropriate treatment of parietal defects have been emphasized as measures to prevent PD failure, discontinuation, and conversion to hemodialysis.^8)^ In addition to the well-known infectious risk of peritonitis, the sustained or recurrent increase in intra-abdominal pressure associated with dialysate dwell may predispose patients to abdominal wall complications, including inguinal hernias.^6,7)^ Early PD dysfunction is often attributed to catheter-related problems.^9)^ Our case highlights hernia-related dialysate sequestration as an additional cause of insufficient intraperitoneal exchange, particularly in patients with prominent inguinoscrotal swelling. Regarding operative strategy, international guidelines for groin hernia management recommend mesh-based repair as the standard approach, with both open anterior (e.g., Lichtenstein) and laparo-endoscopic repair being best evaluated. TEP and TAPP repair have comparable outcomes, and technique selection should be based on the surgeon’s expertise and available resources.^10)^ The guideline summary specifically states that in certain contexts such as patients on peritoneal dialysis, an anterior approach should be considered, reflecting concerns about peritoneal violation and dialysate leakage.^10)^ Although open anterior mesh repair, such as Lichtenstein, is widely recommended and often considered in patients on PD,^10)^ we selected a posterior extraperitoneal approach for several reasons. First, the hernia was large with scrotal extension. An anterior approach would have probably required a larger incision and wider subcutaneous dissection of the inguinal region, potentially increasing the risk of wound-related complications such as infection or seroma. Second, our primary objective was early resumption of PD. TEP provides posterior mesh reinforcement without entering the peritoneal cavity, and is performed through small incisions, which may be advantageous when early postoperative increases in intra-abdominal pressure are anticipated. Additionally, unlike TAPP repair, TEP does not require a peritoneal incision or subsequent peritoneal closure. Moreover, because TEP is performed without transabdominal access to the peritoneal cavity, it prevents abdominal wall penetration into the peritoneal space and may theoretically reduce the risk of port-site dialysate leakage. Therefore, theoretically, TEP may reduce the risk of postoperative dialysate leakage and facilitate earlier resumption of PD when clinically indicated. Finally, given the marked preperitoneal edema observed intraoperatively, meticulous dissection was performed to avoid peritoneal breach while maintaining an extraperitoneal plane suitable for mesh placement. In addition, distal sac transection was selected to minimize the traction of the edematous tissues because the hernia sac was extremely large. The proximal stump was securely double-ligated to reduce the risk of postsurgery leakage into the scrotum. Taken together, TEP appears to offer the most reasonable balance between maintaining peritoneal integrity and minimizing wound morbidity to preserve PD. Therefore, in patients, the surgical objective is not only hernia repair but also preservation of peritoneal integrity to facilitate safe continuation of PD. Although there is no universal consensus on perioperative PD regimens after hernia surgery, many protocols recommend temporarily withholding PD (often 24–48 hours) followed by gradual reintroduction using low-volume exchanges—typically in the supine position and with a “dry day” strategy—to minimize intra-abdominal pressure during early wound healing.^11–13)^ In the present case, because continuing PD was the principal goal and we avoided peritoneal breach, PD was restarted on POD 1 with stepwise volume escalation, returning to the usual regimen by POD 5 without early leakage or recurrence during the 6 months follow-up period. This finding supports the concept that, in selected patients, semi-urgent TEP can be an effective bridge to maintain PD without interim hemodialysis, provided that peritoneal injury is avoided and postoperative PD is carefully titrated and monitored.^11)^

This case has additional implications for PD planning. When a sizable inguinal hernia is known before PD initiation, early elective repair (or synchronous repair at the time of PD access creation, depending on local practice) should be considered to prevent PD dysfunction and urgent surgical intervention.^8)^ Conversely, once severe PD failure occurs due to hernia-related dialysate sequestration, prompt surgical repair may be the only practical solution to restore PD feasibility. In such situations, an extraperitoneal approach, such as TEP, may be particularly advantageous when early PD resumption is desired.

This report is limited by its single-case design, which precludes generalization regarding the optimal surgical approach or postoperative PD regimen. And follow-up was relatively short at 6 months. Larger studies are needed to clarify patient selection and standardized perioperative PD protocols after hernia repair in PD patients.

## CONCLUSIONS

A large inguinal hernia can cause profound PD dysfunction through dialysate sequestration within the hernial sac, even in the absence of catheter migration. Careful pre-PD screening and heightened vigilance after PD initiation are warranted in patients with inguinal hernia. The elective repair of pre-existing inguinal hernias, even if asymptomatic, may be required when PD is planned to reduce the risk of PD interruption or failure and avoid unplanned semi-urgent intervention. By avoiding peritoneal entry, semi-urgent TEP repair may facilitate early postoperative PD resumption and preserve PD as a renal replacement modality.
